# Correlation assessment of SARS-CoV-2 variants and their subvariants present in clinical and wastewater samples in Oregon, USA (February 7, 2021 - February 26, 2022) using the Freyja bioinformatics approach

**DOI:** 10.1371/journal.pone.0357591

**Published:** 2026-09-09

**Authors:** Anirudh Bhatia, Justin Elser, Steven J. Carrell, Brent Kronmiller, Melissa Sutton, Christine Kelly, Tyler S. Radniecki

**Affiliations:** 1 School of Chemical, Biological and Environmental Engineering, Oregon State University, Corvallis, Oregon, United States of America; 2 Center for Quantitative Life Sciences, Oregon State University, Corvallis, Oregon, United States of America; 3 Public Health Division, Oregon Health Authority, Portland, Oregon, United States of America; Emory University, UNITED STATES OF AMERICA

## Abstract

**Background:**

Wastewater surveillance is a valuable tool for monitoring SARS-CoV-2 at the community level. As the virus diversified into many variants and subvariants that share overlapping mutations, resolving them accurately from wastewater becomes a key bioinformatic challenge.

**Objectives and aims:**

This study evaluated two distinct bioinformatic approaches, multilocus sequence typing (MLST) and Freyja, for identifying SARS-CoV-2 variants and subvariants in Oregon wastewater samples collected from February 2021 to February 2022.

**Methods:**

The MLST approach identified SARS-CoV-2 variants using unique mutations curated from clinical samples. In contrast, the Freyja approach resolved variant and subvariant abundances using genome wide mutation profiles weighted by sequencing depth. In this study, the variant and subvariants relative abundances produced by both approaches were compared against those observed in clinical surveillance data.

**Results:**

Both approaches identified SARS-CoV-2 variants at relative abundances that agreed closely with those observed in clinical surveillance data. However, only the Freyja approach identified over 200 Delta subvariants, divided into three clades (21A, 21I and 21J) and two levels (Level 1 and 2) based on Pango subvariants. Delta subvariants showed strong agreement at Level 1 subvariants (r_s_ = 0.892–0.944), while agreement at Level 2 subvariants was inconsistent (r_s_ = 0.324–0.903).

**Conclusions:**

The Freyja approach provided enhanced resolution of SARS-CoV-2 variants and subvariants in wastewater, at abundances that agreed with clinical surveillance. This added resolution is a critical advantage for public health surveillance as SARS-CoV-2 continues to evolve and share mutations across variants and subvariants.

## Introduction

Wastewater comprises a complex blend of diverse biomarkers that provide insights into a wide range of human activities. Wastewater surveillance has been used to monitor the use of illicit drugs and other substances by the community and assess the communal burden of pathogens, including polio, SARS-CoV-2, influenza, respiratory syncytial virus and measles [1–5]. Wastewater surveillance has the benefit of encompassing both symptomatic and asymptomatic individuals and includes those individuals who have not undergone testing [6]. Additionally, wastewater surveillance can identify the distributions of SARS-CoV-2 variants within a community [7,8].

To monitor the rapid acquisition of mutations in the SARS-CoV-2 virus during the COVID-19 pandemic and investigate the impact of genetic variants on disease severity, bioinformatic approaches that include variant calling tools were used to identify low-frequency mutations found in raw sequence data from wastewater samples [7–11]. One of these bioinformatic approaches was the multilocus sequence typing (MLST) approach. The MLST approach was originally used to identify bacterial strains by sequencing multiple housekeeping genes [12]. At each gene locus, all unique sequences identified across a bacterial species are assigned distinct allele numbers, and the combination of allele numbers across all loci defines a sequence type (ST) that serves as a standardized identifier for strain classification and epidemiological tracking [12–14].

When adapted for SARS-CoV-2 genomic surveillance in wastewater, rather than housekeeping genes, the MLST approach sets lineage-defining mutations identified from clinical sequences are matched to mutations detected at multiple genomic loci in wastewater samples to identify and quantify circulating variants [11,15,16]. In addition to identification, the MLST approach has demonstrated strong correlations between SARS-CoV-2 variant relative abundances in wastewater and clinical data [15]. However, the MLST approach was unable to identify subsequent subvariants within SARS-CoV-2 lineages in complex wastewater samples.

In contrast, the Freyja approach can estimate the relative abundances of SARS-CoV-2 variants and their subvariants in wastewater samples by utilizing a collection of unique markers representing different lineages of the SARS-CoV-2 phylogenetic tree [17–19]. This approach examines the frequency of single nucleotide variants (SNVs) within each unique marker, representing a specific mutation in a particular lineage. Furthermore, Freyja considers how often distinct parts of the genomes have been sequenced (*i.e.*, sequencing depth). By taking both sequencing depth and the presence of SNVs into account and by utilizing depth-weighted least absolute deviation-based statistical methods, Freyja achieves an estimation of the relative abundance of each variant and subsequent subvariants in a sample [20]. Additionally, the Freyja approach has been shown to have superior performance relative to other variant identification tools, with improvements in detection accuracy and computational efficiency [9,17,20–23].

However, the extent to which SARS-CoV-2 subvariants identified in wastewater by the Freyja approach correspond to those identified in GISAID clinical submissions has yet to be thoroughly examined [24]. In this study, the relative abundances of SARS-CoV-2 variants in Oregon wastewater identified with the Freyja approach were correlated with the relative abundances of SARS-CoV-2 variants in Oregon wastewater previously identified by the MLST approach [15]. Additionally, the relative abundances of SARS-CoV-2 variants and subsequent subvariants found in Oregon wastewater samples with the Freyja approach were correlated with the relative abundances of SARS-CoV-2 variants and subsequent subvariants identified in Oregon clinical samples submitted to GISAID. The subvariant correlation focused on all clades of Delta variants present during the study period from February 7, 2021 – February 26, 2022.

## Materials and methods

### Wastewater collection, concentration and RNA extraction

**Fig 1:**
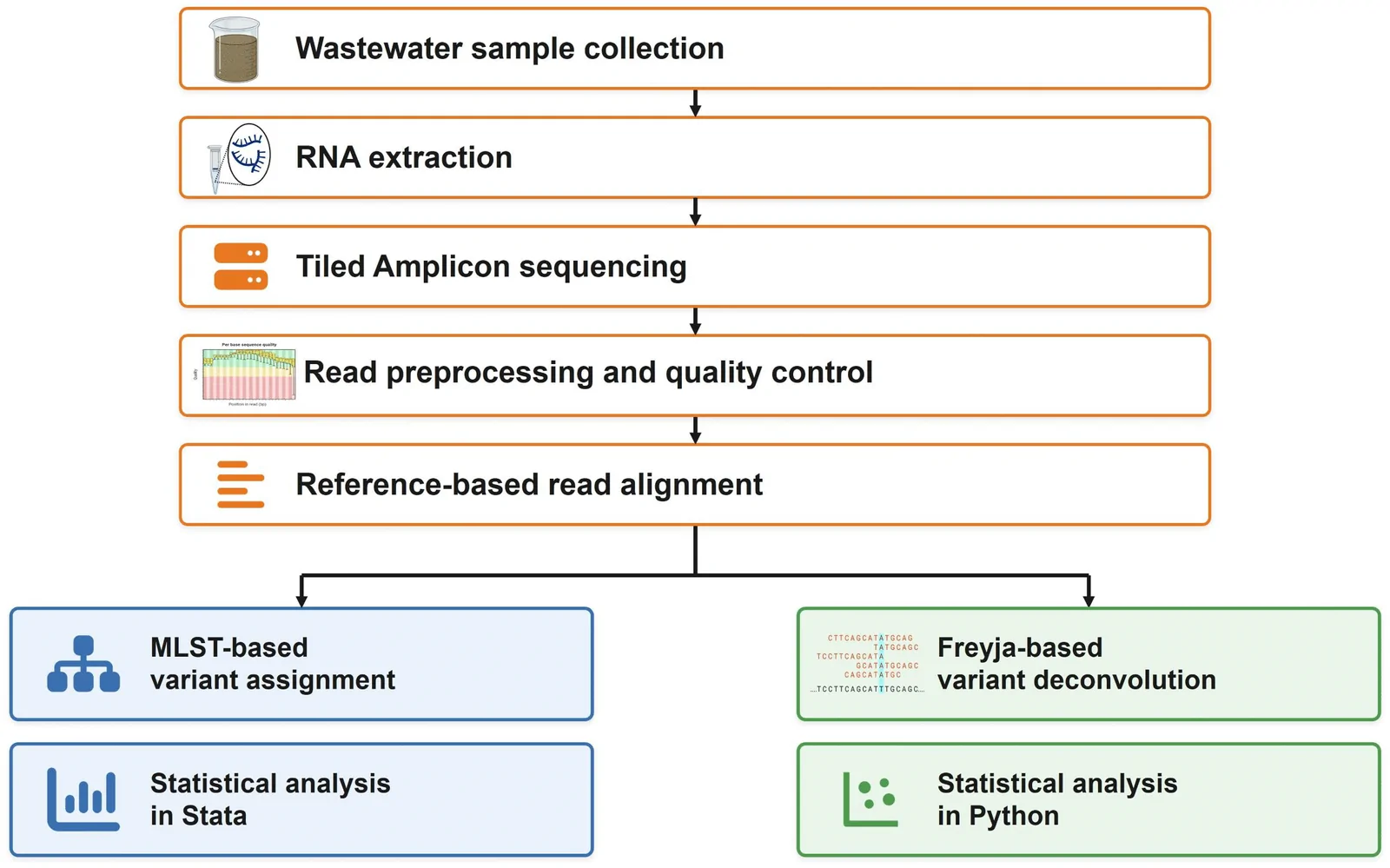
Workflow for comparative relative abundance of SARS-CoV-2 variants/subvariants in wastewater and clinical samples.

From February 7, 2021, through February 26, 2022, 24-hour composite samples were collected > 1 time per week from the influents of up to 43 Oregon-based wastewater treatment facilities (Fig 1). Wastewater samples were collected from municipal wastewater treatment facilities participating in the Oregon statewide SARS-CoV-2 wastewater surveillance program, a public health surveillance effort coordinated by the Oregon Health Authority and Oregon State University. Sample collection was conducted with the knowledge and permission of each participating utility as part of their voluntary participation in this program. No specific permits were required for this study. The Oregon State University Institutional Biosafety Committee determined wastewater surveillance is exempt from the human subjects regulations set forth by the U.S. Department of Health and Human Services (45 CFR 46). All the clinical sequencing data were publicly available on GISAID.

The samples were vacuum-filtered onsite using a 0.45-µm, 47-mm diameter mixed cellulose ester electronegative filter which was subsequently placed into 1 mL of DNA/RNA Shield (Zymo Scientific, USA) for RNA stabilization. The stabilized filters underwent bead beating with 0.7-mm garnet beads for 2 minutes, and RNA was extracted using the MagMAX Viral/Pathogen Nucleic Acid Isolation Kit (Thermo Fisher Scientific). SARS-CoV-2 RNA concentrations were quantified using reverse transcription droplet digital PCR (RT-ddPCR) on a QX200 ddPCR system (Bio-Rad Laboratories, Hercules, CA) using the 2019-nCoV CDC ddPCR Triplex Probe Assay and One-Step RT-ddPCR Advanced Kit protocol [11,15].

Amplicon libraries were generated using the Swift Amplicon SARS-CoV-2 Panel and Swift Amplicon Combinatorial Dual indexed adapters (Integrated DNA Technologies [IDT] Swift Biosciences). Sequence reads from 2161 samples were preprocessed and demultiplexed with zero index mismatches using bcl2fastq2 version 2.20 for HiSeq 3000 and BCL Convert version 1.2.1 for NextSeq 2000. Reads were trimmed using the BBDuk tool (BBMap version 38.84 (US Department of Energy Joint Genome Institute, https://jgi.doe.gov)) and aligned to reference sequence (Wuhan-Hu-1, GenBank accession no. NC_045512.2) using the BWA-MEM algorithm version 0.7.17-r1188 (https://github.com/lh3/bwa). The reads were coordinate sorted using SAMtools version 1.10, and the primer sequences were removed using Primerclip version 0.3.8 [25,26]. SAMtools were also used to convert SAM files to BAM files and to coordinate and sort the BAM files [25]. The sequencing reads were submitted to NCBI and are present under Bioproject: PRJNA938474 [27].

### Identification of SARS-CoV-2 variants using the Multilocus Sequence Typing (MLST) approach

The Genome Analysis Toolkit (GATK) was utilized to identify mutations in the sequence reads by comparing the reads against the Wuhan-Hu-1 reference genome [28]. Within the GATK toolkit, HaplotypeCaller was used to call variants on a per-sample basis, followed by joint genotyping across all samples using CombineGVCFs and GenotypeGVCFs, and final conversion of the resulting VCF file into a tabular format using VariantsToTable for downstream MLST-based analysis [11,29]. The Integrative Genomics Viewer (IGV) was used to manually inspect sequence alignments and confirm mutation calls, ensuring accuracy in mutation detection [30]. The MLST approach was applied to classify the mutations into known SARS-CoV-2 variants. To screen specific mutations unique to each variant, the mutations found in the wastewater samples were compared against a comprehensive database of mutations from clinical specimens and published variant data. The final identification of a variant in a wastewater sample was based on two criteria: 1) a minimum threshold of 5% of sequence reads with at least 6 total reads covering the mutation site, and 2) at least 2 different sites carrying a variant mutation must be present [11,16].

### Identification of SARS-CoV-2 variants and subvariants using the Freyja approach

The same sequence reads that were classified by the MLST approach were also classified using the Freyja approach. Applying both approaches to identical sequencing reads ensured that any differences in the variants and relative abundances reported were attributable to the bioinformatic approach rather than to variation in sample processing or sequencing. The Freyja approach identified SARS-CoV-2 variants by linking variants to site-specific SNPs, which act as unique mutation signatures, derived from a global phylogenetic tree created by UShER [18]. The Freyja approach utilized a depth-weighted least absolute deviation regression approach to deconvolve the relative abundances of identified variants [20]. This method emphasized data from regions of the genome with higher sequencing depth to provide more reliable mutation frequency estimates [17]. The analysis was constrained so that the sum of all lineage abundances equals one and each abundance value must be non-negative [23,31,32]. After identification and relative abundance calculations were completed, the variants and subvariants were classified as SARS-CoV-2 variants and subvariants according to Pango nomenclature [33]. Variant calling and demixing were performed using Freyja version 1.4.5 with the UShER barcode library dated 31 July 2023.

### Comparison of variant relative abundances identified with the MLST and Freyja approaches

For consistency in labeling data across genomic surveillance studies and to facilitate comparisons between the MLST and Freyja protocols, an in-house Python notebook (https://github.com/AnirudhB7/Freyja_Correlation_Study) was used to group the variants and subvariants, as defined by the World Health Organization nomenclature, using a Pango lineage to WHO variant mapping retrieved in January 2024 from the Nextclade SARS-CoV-2 dataset (https://nextstrain.org/nextclade/nextstrain/sars-cov-2/wuhan-hu-1/orfs) [34,35]. The mapping file used is available in the code repository. For instance, variants and subvariants identified as BA.2 and BA.2.86, using Pango nomenclature, were grouped under the Omicron variant as named using WHO classification system. Once grouped, the weekly (based on epi weeks) state-wide variant relative abundances using either GISAID (https://www.gisaid.org/) (Equation 1) or wastewater (Equation 2) data were calculated [24].


$$\begin{array}{c} S_{c}=\frac{n}{N} \end{array}$$ (1)

$S_{c}$ = *Statewide relative abundance of a variant or subvariant in clinical samples,*

n = *Number of clinical samples belonging to a specific variant (or subvariant) reported for each epiweek,*

N = *Total number of clinical sequence samples reported for that epiweek*


$$\begin{array}{c} S_{w}=\frac{\sum j(C_{v,j}\;\times \;F_{j})}{\sum j\sum i(C_{vi,j}\;\times \;F_{j})} \end{array}$$ (2)

$S_{\text{w}}$  = *Statewide relative abundance of a variant or subvariant in wastewater,*

$C_{v}=\frac{Gene\;copies\;(gc)}{L}$ *for each variant in each location,*

F *= Flow rate of that location on a given day,*

$\frac{gc}{d}v=\frac{Gene\;copies}{day}\;$ *reported for each variant* (v) *on a given day =* $C_{v}*F$,

$\frac{gc}{d}total$ *= Total gene copies for all variants recorded on a given day =* $\sum \;{C_{v}}_{i}\;*\;F$,

i  *= all variants in the sample,*

j = all locations included in a particular epiweek,

$C_{v,j}$ = *wastewater SARS-CoV-2 variants concentration at location j,*

$F_{j}$  *= wastewater flow rate for location j.*

The relative abundances of the SARS-CoV-2 variants from the MLST and Freyja approaches were organized into spreadsheet-like data frames using Python’s Pandas package (https://pandas.pydata.org). Additionally, the variant relative abundances identified in clinical and wastewater samples by the Freyja approach were also organized into a separate data frame. This organization facilitated a clear comparison between the two datasets and streamlined subsequent statistical analyses.

All statistical analyses were conducted using Python’s SciPy package (https://scipy.org). Normality of the data was assessed via the Shapiro-Wilk test using the ‘shapiro’ function from the SciPy package. With the normality test indicating that the data did not follow normal distribution, Spearman’s rank correlation analyses were conducted on the data sets using the ‘spearmanr’ function in the SciPy package. Since weekly observations within an epidemic wave are serially dependent, so the number of effectively independent observations is smaller than the number of weeks sampled and conventional significance tests for rank correlation are not appropriate. Nominal p-values were therefore not used. Uncertainty in each correlation coefficient was instead quantified using a block bootstrap, an approach developed for serially correlated data [36], implemented with the CircularBlockBootstrap function of the Python arch package (https://arch.readthedocs.io/en/latest/bootstrap/timeseries-bootstraps.html). Blocks of four consecutive weeks were used, following the n^(1/3) rate for block length selection with n = 55 weekly observations [36]. Each 95% confidence interval is based on 2,000 replicates with a fixed random seed. Confidence intervals that exclude zero indicate agreement between the two measures, with the lower bound giving a conservative estimate of its strength. Intervals that include zero indicate that the data are consistent with no association and are not interpreted as evidence of agreement.

Time-series plots comparing the MLST and Freyja approaches as well as the variant trends in wastewater and clinical samples were built using Python’s matplotlib package (https://matplotlib.org). Correlation panels include an identity line (y = x) as a reference for agreement between the two measures, with both axes on a common scale within each panel.

### Correlation of Delta ‘AY’ lineage variant and subvariant relative abundances identified in wastewater and clinical cases

The relative abundance data of Delta ‘AY’ variants (*e.g.*, AY.26) and subvariants (*e.g.*, AY.26.1) found in wastewater were identified using the Freyja approach and clustered according to Pango nomenclature. These classifications were mapped onto Nextstrain nomenclature system and clades were identified [37]. Metadata for all publicly available Delta ‘AY’ variants and subvariants were retrieved from the Nextstrain database, which provided information about their Pango nomenclature and the corresponding clade categories assigned to them [35].

**Fig 2:**
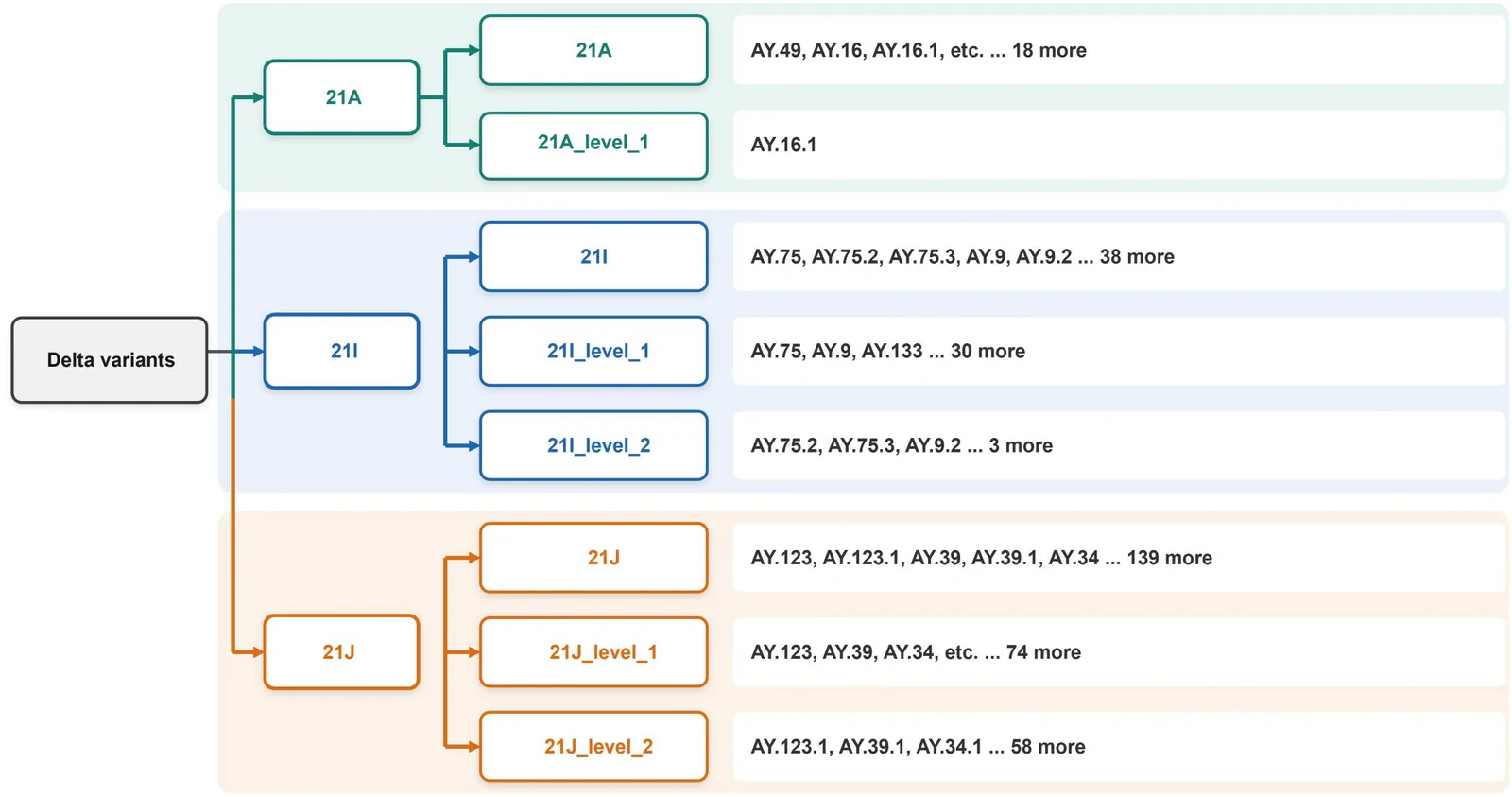
Classification of Delta variants and subvariants categorized using Nextstrain clade nomenclature.

Subvariants within each Delta clade were categorized based on the number of periods in their Pango nomenclature. For example, SARS-CoV-2 variants AY.16 and AY.16.1, as identified on the NextStrain database, are part of clade 21A. In this instance, the relative abundances of AY.16 and AY.16.1 in wastewater samples were aggregated under the Delta_21A category. For finer resolution, AY.26 was distinguished from its sub lineage AY.26.1 based on the PANGO lineage hierarchy and AY.16 was specifically included in Delta_21A_Level_1 and AY.16.1 in Delta_21A_Level_2. This classification approach is consistently applied to clades 21I and 21J (Fig 2).

Similarly, the subvariants belonging to each Delta clade in GISAID clinical samples were categorized by downloading data from the Nextstrain database, which contained data related to the variants/subvariant classifications (*e.g.*, their Pango nomenclature), their WHO lineage (*e.g.*, Delta) and their corresponding clade name (*e.g.*, 21A, 21I and 21J). The GISAID clinical samples were filtered by variant to isolate Delta-containing samples. The identified Delta variants/subvariants were segregated by clade (*i.e.*, 21A, 21I and 21J) and sorted by epiweek.

Once segregated into clades, the samples were further categorized by level (*e.g.*, Delta_21A_Level_1, Delta_21A_Level_2, Delta_21I_Level_1, Delta_21I_Level_2, Delta_21J_Level_1 and Delta_21J_Level_2). This was done to maintain consistency of the categorizing between variants recognized in wastewater and clinical samples. Relative abundances at the variant, subvariant and level categories for both wastewater and clinical samples were calculated as described above (Equations 1 and 2). The normality of the subvariant distributions were evaluated using the Shapiro-Wilk test, as described above. As both the wastewater and clinical subvariants within each Delta clade was non-normally distributed in either sample type, Spearman’s rank correlation analyses were conducted and the time series data was plotted, as described above.

## Results

### Temporal distribution of SARS-CoV-2 variant relative abundances in clinical and wastewater samples with the Freyja and MLST approaches

**Fig 3:**
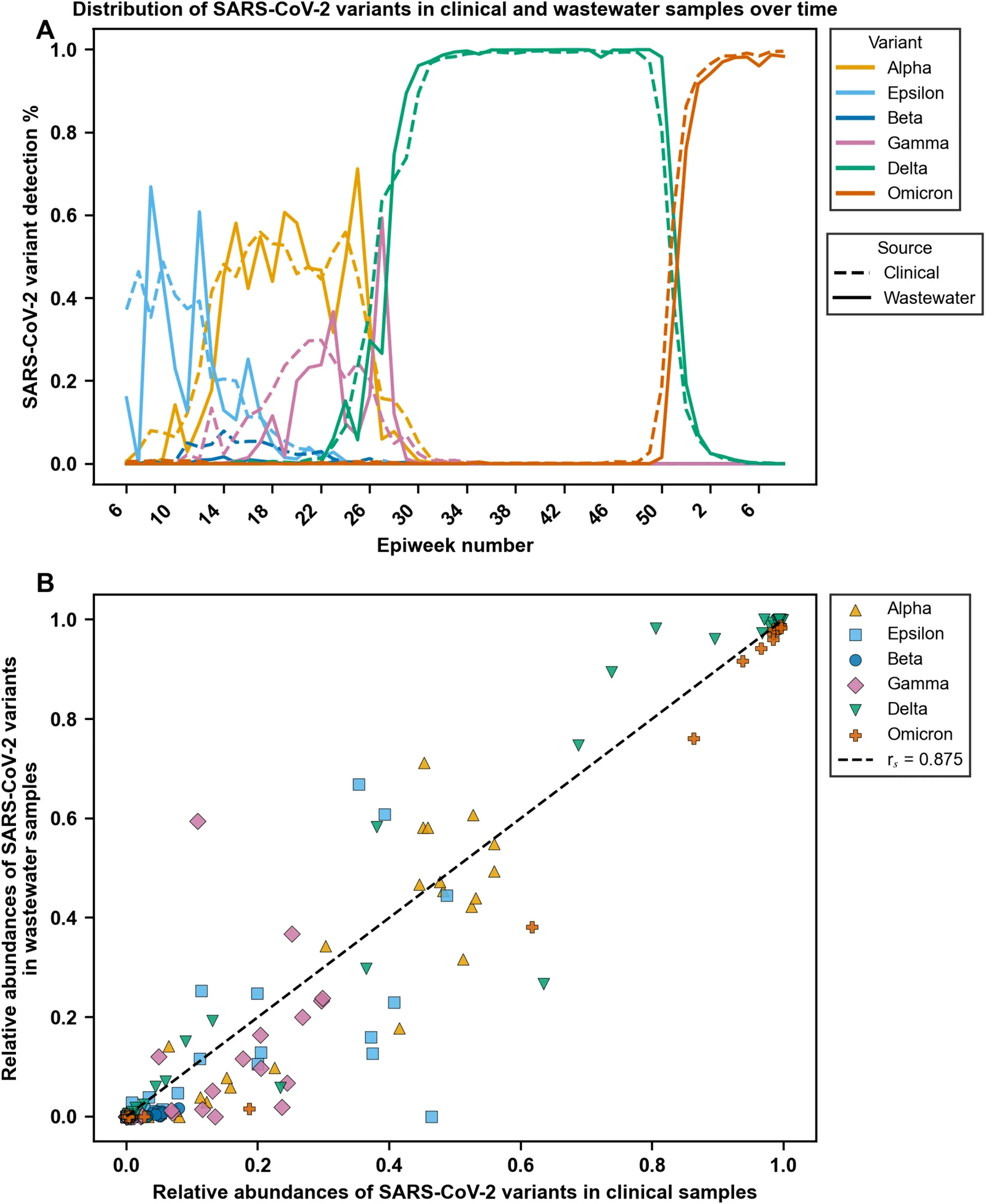
Comparative analysis of SARS-CoV-2 variant abundances in wastewater and clinical genomic surveillance in Oregon, USA. (**a**) Distribution of SARS-CoV-2 variant abundances in clinical specimens and wastewater samples collected in Oregon between February 6, 2021, and February 26, 2022. (**b**) Correlation plot of SARS-CoV-2 variant abundances in clinical and wastewater datasets.

**Fig 4:**
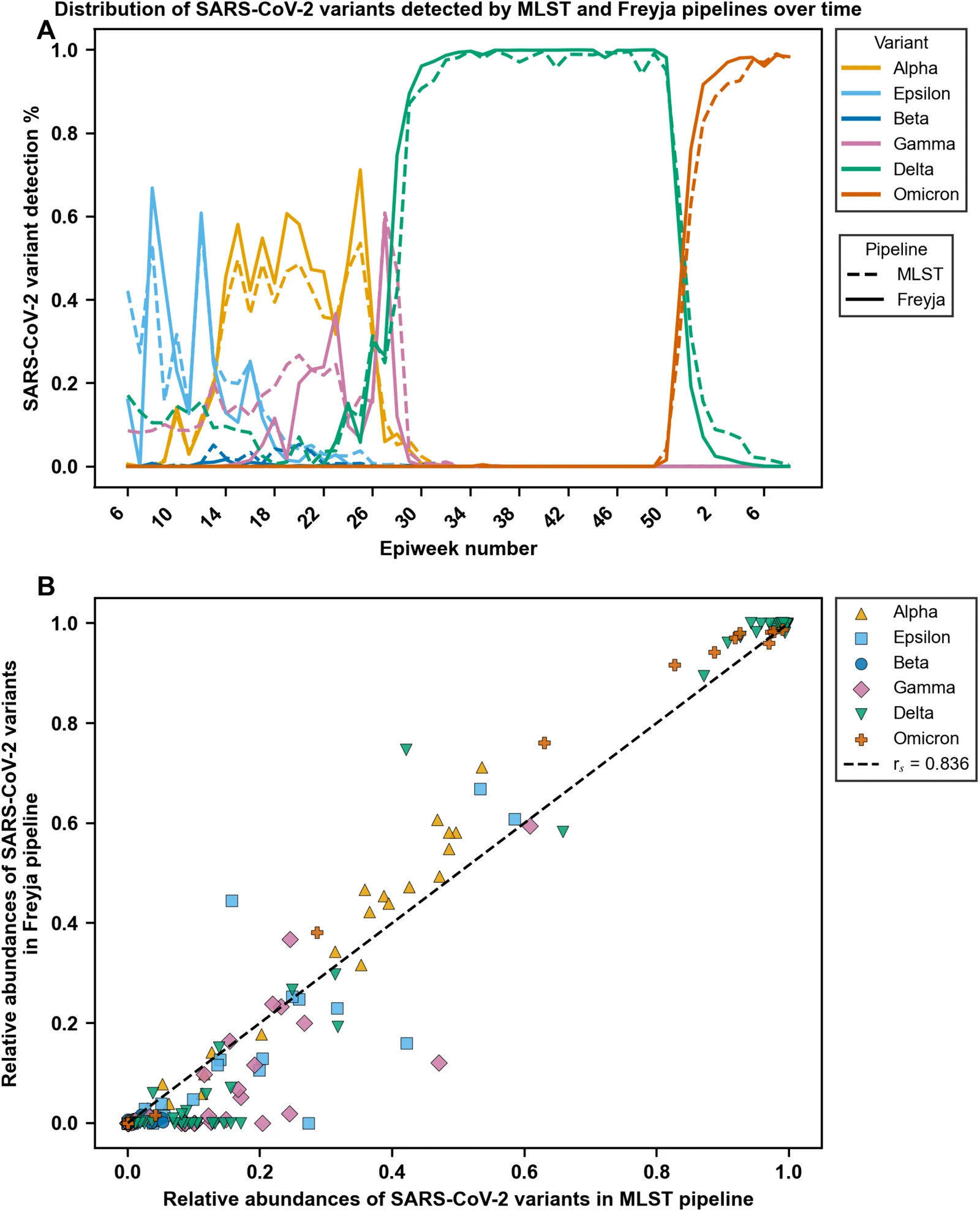
SARS-CoV-2 variant detection using the MLST and Freyja based pipelines in Oregon wastewater samples. (**a**) Weekly relative abundances of severe acute respiratory syndrome coronavirus 2 (SARS-CoV-2) variants detected in wastewater samples collected in Oregon between February 6, 2021, and February 26, 2022 using the multi-locus sequence typing (MLST) and the Freyja tool. (**b**) Correlation plot of SARS-CoV-2 variants detected in wastewater samples analyzed by the MLST and Freyja pipelines.

Over the 55-week study period, SARS-CoV-2 variants emerged, peaked, and declined synchronously in both the clinical and wastewater samples analyzed by the Freyja approach (Fig 3a). The relative abundances of SARS-CoV-2 variants in clinical and wastewater samples showed strong agreement, with a strong positive correlation (r_s_  = 0.875) (Fig 3b). Similarly, the wastewater SARS-CoV-2 variant relative abundances identified by the Freyja approach showed a high degree of agreement with those identified by the MLST approach (Fig 4a). The variant relative abundances identified by both approaches showed strong concordance with one another (r_s_ = 0.836) (Fig 4b), indicating that the Freyja approach reproduced the variant-level results of the established MLST approach on the same sequencing data.

The exceptions to the strong similarity between the Freyja and MLST results were the Beta and Gamma relative abundances. The Beta and Gamma variants showed the weakest agreement between the two approaches within wastewater samples (r_s_ = 0.704 and 0.772) (**Table 1**). This weaker agreement between the two approaches for both Beta and Gamma variants may be due to broad range of subvariants analyzed by the Freyja approach (5 for Beta and 20 for Gamma), compared to the single subvariant analyzed by the MLST approach for both Beta and Gamma. Additionally, the higher sensitivity of the Freyja approach, which considers a wider set of mutations for variant calling compared to the MLST approach, which only focuses on specific locus for calling a mutation, may also have led to differences in the reported relative abundances of the Beta and Gamma variants.

**Table 1:** Statistical assessment of SARS-CoV-2 variant relative abundances between the MLST and Freyja bioinformatic approaches in wastewater samples.

Variant	r_s_	CI (95%)
All	0.836	0.736 - 0.915
Alpha	0.911	0.686 - 0.981
Epsilon	0.839	0.612 - 0.950
Beta	0.704	0.278 - 0.865
Gamma	0.772	0.407 - 0.933
Delta	0.810	0.491 - 0.926
Omicron	0.966	0.789 - 1.000

Overall, the modest disagreements between the SARS-CoV-2 variant relative abundances identified by the MLST or Freyja approaches may be due to differences in how each approach calls variants. When compared to the relative abundances of SARS-CoV-2 variants in clinical samples, the MLST approach had slightly higher degree of correlation with clinical data for earlier SARS-CoV-2 lineages, such as Alpha and Epsilon (**Table 2**). However, later lineages, including Beta, Gamma, and Delta, increased in mutational diversity and an increased number of subvariants observed. Under these conditions, the Freyja approach showed either equivalent (in case of Gamma variant) or higher degree of correlation to clinical variants.

**Table 2:** Statistical assessment of SARS-CoV-2 variant relative abundances of the MLST and Freyja bioinformatic approaches between wastewater and clinical samples.

Variant	r_s__MLST	r_s__Freyja	CI (95%)_MLST	CI (95%)_Freyja
All	0.812	0.875	0.688 - 0.900	0.813 - 0.918
Alpha	0.942	0.932	0.808 - 0.975	0.784 - 0.976
Epsilon	0.907	0.883	0.710 - 0.967	0.703 - 0.987
Beta	0.774	0.832	0.423 - 0.895	0.590 - 0.940
Gamma	0.875	0.873	0.652 - 0.958	0.650 - 0.962
Delta	0.818	0.943	0.457 - 0.931	0.804 - 0.976
Omicron	0.699	0.657	0.326 - 0.886	0.200 - 0.885

For both the MLST and Freyja approaches, the Omicron variant had the lowest degree of agreement between the wastewater and clinical samples (rs = 0.699 and 0.657). This result could be due to the limited number of Omicron samples included in this study, which may not have captured the overall trend for this lineage when compared to clinical samples.

The stronger agreement observed with the Freyja approach for the Beta and Delta variants reflected the differences in their underlying variant complexity and abundance. For example, the Delta variant was comprised of about 201 AY subvariants which signifies substantial mutational heterogeneity. As the number of subvariants increases, fewer mutations remain uniquely informative for lineage assignment. This effectively limits locus-based approaches such as MLST.

In the case of Beta variants, they were never the dominant variant during the study period (Fig 3a and 4a). Thus, the mutations which were initially characteristic of the lineage may have been shared with or co-opted by other circulating variants which increases the potential for misclassifying by the MLST approach. Thus, these observations suggest that while both approaches perform well overall, the Freyja approach provides more robust variant abundance estimates for lineages characterized by low abundance and extensive subvariant diversity and showed an overall higher correlation with clinical data compared to the MLST approach.

### Relationship between the relative abundances of SARS-CoV-2 Delta AY subvariants identified in clinical samples and wastewater samples analyzed by the Freyja approach

The performance of the Freyja approach in resolving subvariants was assessed by comparing the relative abundances of Delta AY subvariants in wastewater with those observed in clinical samples. The Delta AY variant was specifically chosen due to its large subvariant population with 201 known Delta AY subvariants that are categorized into three clades (21A, 21I and 21J) based on Nextstrain nomenclature (Fig 2). The large number of Delta AY subvariants resulted in many Delta AY subvariants sharing mutations, which led to very few wholly unique mutations for each Delta AY subvariant. This lack of wholly unique mutations provided difficulties for the MLST approach to accurately identify the Delta AY subvariants.

The sharing of unique mutations between closely related Delta AY subvariants did not limit the effectiveness of the Freyja approach in identifying Delta AY subvariant relative abundances. This was quantified through the strong correlations observed between the clinical samples and Delta AY subvariant relative abundances calculated by the Freyja approach for the Delta AY variant overall (r_s_ = 0.923), the three clades of Delta 21A, 21I and 21J (r_s_ = 0.892–0.959), and the three Level 1 sub-clades (r_s_ = 0.892–0.944) (**Table 3**). However, for the Level 2 sub-clades, the agreement between the wastewater and clinical relative abundances began to decline.

**Table 3:** Statistical assessment of SARS-CoV-2 Delta subvariant relative abundances of the Freyja bioinformatic approaches between wastewater and clinical samples.

Subvariants	r_s_	CI (95%)
All	0.923	0.832 - 0.963
Delta_21A	0.899	0.718 - 0.963
Delta_21A_level_1	0.899	0.715 - 0.964
Delta_21I	0.892	0.725 - 0.958
Delta_21I_level_1	0.892	0.729 - 0.960
Delta_21I_level_2	0.324	−0.074 - 0.699
Delta_21J	0.959	0.876 - 0.983
Delta_21J_level_1	0.944	0.824 - 0.975
Delta_21J_level_2	0.903	0.695 - 0.951

**Fig 5:**
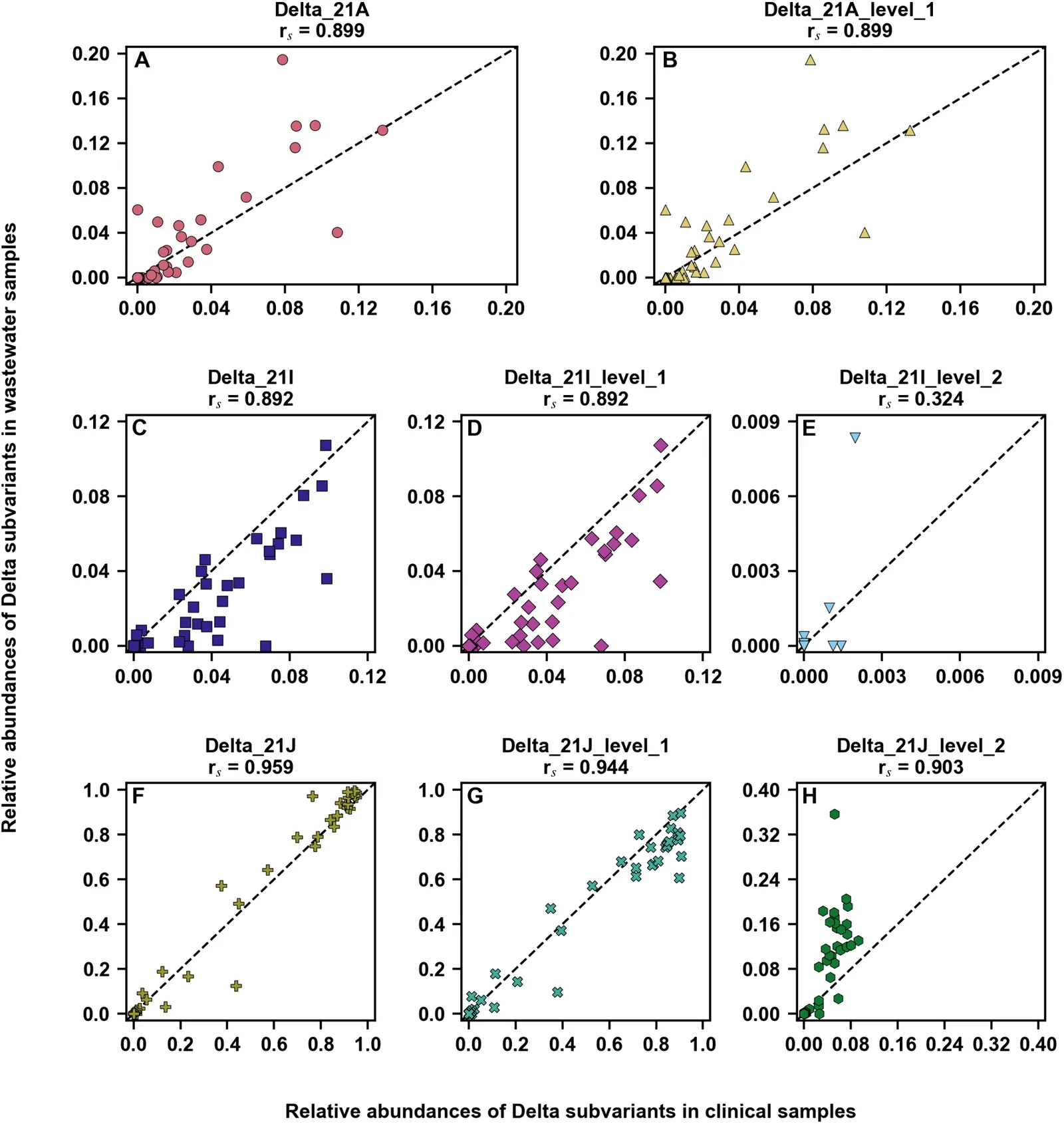
Correlation of Delta subvariant relative abundances in wastewater and clinical surveillance in Oregon, USA.

For instance, the Delta 21J Level 2 sub-clade retained strong agreement (r_s_ = 0.903), whereas the Delta 21I Level 2 sub-clade showed a much weaker association (r_s_ = 0.324) (Fig 5). This outcome is due to the low relative abundances and infrequent detection of the Delta 21I Level 2 subvariant in wastewater samples, as well as the absence of corresponding Delta 21I Level 2 subvariant detections in clinical data. The Delta 21I Level 2 subvariant was detected in 5 of the 55 study weeks in the clinical dataset and 6 of 55 in the wastewater dataset, with detections coinciding in only 2 weeks. The confidence interval for this comparison included zero (95% CI −0.074 to 0.699), and the correlation is therefore not interpreted as evidence of agreement.

For Delta 21J Level 2 variants, the agreement between clinical and wastewater samples remained high (r_s_ = 0.903), though it was lower than when compared to the Delta 21J Level 1 subvariant (r_s_ = 0.944). This was due to relatively few Delta 21 J Level 2 subvariants identified in the clinical samples compared to wastewater samples (**Table 3**). Finally, the Freyja approach identified the presence of Level 3 subvariants in Delta clades J (AY.25.1.1) in five wastewater samples collected from December 20, 2021 – December 27, 2021. However, this study did not extend to Level 3 subvariants due to the lack of clinical data. Therefore, the agreement at this resolution could not be assessed. Thus, this work demonstrated that the Freyja approach recovered Delta subvariant relative abundances in wastewater that agreed with clinical surveillance for Level 1 and, except for the 21I, Level 2 subvariants.

## Discussion

This study compared and contrasted the capabilities of two bioinformatic approaches, MLST and Freyja, to quantify the relative abundances of SARS-CoV-2 variants and subvariants in wastewater samples. These approaches can be broadly categorized into two functional components. The first component is variant calling, which involves mutation detection at each individual base position from sequencing reads. A wide variety of variant callers have been commonly employed in SARS-CoV-2 wastewater surveillance including GATK HaplotypeCaller, iVar, LoFreq, FreeBayes, VarScan, ShoRAH, Clair3 and BCFtools [9,11,16,17,28,29,38–43].

The second component of the bioinformatic approaches is variant detection and quantification. This component is critical in translating the mutations detected by variant calling into meaningful epidemiological information, including variant identification and their relative abundances. Methods developed for this purpose include COJAC, lineagespot, the constrained linear regression model, Kallisto, VaQuERo, LolliPop, LCS (Lineage deComposition), MLST and Freyja [9,11,16,17,21,22,31,32,38,43–47].

In this study, the Freyja approach successfully identified SARS-CoV-2 variants and their relative abundances, which had a high degree of correlation with clinical sequence data. This ability to correlate wastewater variant composition and relative abundances with clinical sequence data is in agreement with other studies that have used numerous bioinformatic approaches including, MLST, Freyja, the constrained linear regression model, VaQuERo, and the SARS-CoV-2 mutations analysis tool of the QIAGEN CLC Genomics Workbench [15,31,44,45,48]. The success of such a diverse range of bioinformatic approaches is likely due to the high number of unique mutations carried by each SARS-CoV-2 variant.

However, as the number of unique mutations decreases, as is the case for Level 1, 2 and 3 SARS-CoV-2 subvariants, many bioinformatic approaches struggle to resolve SARS-CoV-2 diversity. For instance, signature mutation-based approaches including the MLST, COJAC, and lineagespot approaches identify variants by matching detected mutations to curated panels of lineage defining mutations [9,11,38]. The COJAC approach requires that multiple signature mutations co-occur on the same sequencing read pair [9]. But this becomes less likely as the signature mutations become fewer and more spread out on the SARS-CoV-2 genome, resulting in the failure of the COJAC approach to identify SARS-CoV-2 subvariants.

Additionally, while the MLST and lineagespot approaches identify variants by matching detected mutations against curated panels of lineage defining mutations, they estimate the relative abundances of the variants from the frequency of those mutations in the sequencing data [11,38]. Since each variant is assessed independently, the resulting abundance estimates do not reflect the true proportional composition of the sample. Furthermore, the accuracy of these estimates is sensitive to sequencing depth, as reliable detection of low-abundance SARS-CoV-2 subvariants (Level 1 and Level 2) requires a minimum coverage of 1,000x with best sensitivity only observed at 10,000x or greater [17,49]. This level of sequence coverage is challenging to achieve in wastewater samples, resulting in the inability to accurately identify and quantify SARS-CoV-2 subvariants.

In contrast, the Freyja approach was developed to move beyond individual variant assessment toward simultaneous estimation of all circulating lineages from genome-wide mutation frequency data, including SARS-CoV-2 subvariants [17,31,32]. To accomplish this, the Freyja approach utilizes the iVar variant caller. In a benchmark study with six variant callers, iVar detected more expected lineage-defining mutations than GATK, BCFtools, FreeBayes, VarScan and LoFreq [39]. Additionally, Freyja’s variant detection and quantification component matches observed mutation frequencies against a reference set of lineage-defining profiles and estimates the proportional contribution of each lineage through a regression-based statistical framework.

The false positive variant calls from the Freyja approach are limited by using a depth-weighted analysis approach which prioritizes sequencing data based on sequencing depth, giving greater values to those regions of the genome that have greater sequence depth [17,19,23]. The Freyja approach also compares the frequency of mutations present in the sample’s sequencing data to the known mutations of viral variants as submitted in the global phylogeny tree UShER. This enables the identification of variants that have not appeared in the clinical sequence databases and allows for the identification of subvariants that have relatively few unique mutations [18].

In this study, utilizing the advantages of the Freyja approach, over 200 Delta subvariants were identified and agreement between wastewater and clinical relative abundances was strong at Level 1 subvariants and variable at Level 2. The Freyja approach also detected Level 3 Delta subvariants in wastewater. However, no agreement was evaluated at the Level 3 Delta subvariants because of unavailability in the clinical data. Agreement was limited by how sparsely these subvariants were represented in the comparison data rather than by the ability of the Freyja approach to resolve them. More generally, the clinical dataset serves as a comparator rather than a ground truth. The Delta AY clades represented a demanding case for subvariant resolution, as their subvariants share the majority of their lineage-defining mutations and leave few wholly unique mutations per subvariant. Contemporary lineages additionally arise through recombination between parental lineages, a mode of descent not represented in the Delta AY clades, and extension of this approach to recombinant lineages warrants separate evaluation.

Other bioinformatic approaches in addition to Freyja have been developed to match observed mutation frequencies or sequencing reads against a reference set of lineage-defining profiles to estimate the proportional contribution of each lineage and may be useful in identifying SARS-CoV-2 subvariants. However, Freyja has distinct advantages over these other approaches. For instance, Kallisto and LCS are computationally intensive, particularly in complex wastewater communities, and Kallisto’s performance is dependent on the reference database [17,22]. These limitations can provide challenges if computational resources are limited and do not enable the identification of novel variants not yet found in clinical sequence databases. Additionally, unlike the Freyja approach, the Kallisto, LCS, VaQuERo, LolliPop, and constrained linear regression model approaches all lack automatic updating of lineage profiles and do not account for sequencing depth during abundance estimations [17,18,23]. These limitations lead to higher false positive detection rates in complex mixed community wastewater matrices.

In conclusion, the implementation of the Freyja approach in this genomic surveillance study significantly enhanced the detection and characterization of SARS-CoV-2 variants and subvariants in wastewater samples. The Freyja approach exceeded the MLST approach in variant resolution and detection capabilities and showed strong agreement with the variant temporal dynamics observed in clinical data down to Level 1 subvariants, with variable agreement at Level 2. Thus, this study demonstrates that the Freyja approach recovers relative abundances of SARS-CoV-2 variants and subvariants in wastewater that agree with those observed in clinical surveillance, making it an effective tool for public health surveillance.

However, the Freyja approach has several limitations. Operationally, it lacks built-in functionalities for generating summary reports, visualization statistics, and conducting geospatial analyses. Lineage assignment is entirely dependent on the UShER global phylogeny and the barcode library derived from it. Lineages absent from the tree at the time of analysis cannot be assigned, and because these resources are updated frequently, the same sequencing data analyzed with barcode files from different dates can yield different subvariant abundance distributions. Another shortcoming is that the amplicon coverage in wastewater samples is frequently uneven, and dropout of individual amplicons removes informative sites from the deconvolution. Because the depth-weighted regression assigns weight according to observed sequencing depth, samples with low or uneven genome coverage may return abundance estimates supported by relatively few informative sites. Resolution of closely related lineages depends on a few discriminating sites, so estimates warrant caution when a lineage is present at low abundance. Nevertheless, Freyja’s sensitivity, relatively low computational requirements and ability to resolve the relative abundances of viral variants and subvariants, compensates for these shortcomings.
